# Abnormal male reproduction and embryonic development induced by downregulation of a phospholipid fatty acid-introducing enzyme Lpgat1 in zebrafish

**DOI:** 10.1038/s41598-022-11002-4

**Published:** 2022-05-04

**Authors:** Takeaki Shibata, Hiroki Kawana, Yuri Nishino, Yoshiko Ito, Hiroyasu Sato, Hirofumi Onishi, Kuniyuki Kano, Asuka Inoue, Yoshitaka Taketomi, Makoto Murakami, Satoshi Kofuji, Hiroshi Nishina, Atsuo Miyazawa, Nozomu Kono, Junken Aoki

**Affiliations:** 1 Department of Health Chemistry, Graduate School of Pharmaceutical Sciences, The University of Tokyo, 7-3-1, Hongo, Bunkyo-ku, Tokyo, 113-0033 Japan; 2 Laboratory of Molecular & Cellular Biochemistry, Graduate School of Pharmaceutical Sciences, Tohoku University, 6-3, Aoba, Aramaki, Aoba-ku, Sendai, Miyagi, 980-8578 Japan; 3 Department of Developmental and Regenerative Biology, Medical Research Institute, Tokyo Medical and Dental University, Tokyo, Japan, 5-45, Yushima, Bunkyo-ku, Tokyo, 113-8510 Japan; 4 Advanced Research & Development Programs for Medical Innovation (AMED-LEAP), Chiyoda-ku, Tokyo, 100-0004 Japan; 5 Graduate School of Science, University of Hyogo, 3-2-1, Kouto, Kamigori-cho, Ako-gun, Hyogo, 678-1297 Japan; 6 Laboratory of Microenvironmental and Metabolic Health Science, Center for Disease Biology and Integrative Medicine, Graduate School of Medicine, The University of Tokyo, 7-3-1, Hongo, Bunkyo-ku, Tokyo, 113-0033 Japan

**Keywords:** Phospholipids, Lipidomics

## Abstract

Phospholipids in the membrane consist of diverse pairs of fatty acids bound to a glycerol backbone. The biological significance of the diversity, however, remains mostly unclear. Part of this diversity is due to lysophospholipid acyltransferases (LPLATs), which introduce a fatty acid into lysophospholipids. The human genome has 14 LPLATs and most of them are highly conserved in vertebrates. Here, we analyzed the function of one of these enzymes, lysophosphatidylglycerol acyltransferase 1 (Lpgat1), in zebrafish. We found that the reproduction of heterozygous (*lpgat1*^+/−^) male mutants was abnormal. Crosses between heterozygous males and wild-type females produced many eggs with no obvious cleavage, whereas eggs produced by crosses between heterozygous females and wild-type males cleaved normally. Consistent with this, spermatozoa from heterozygous males had reduced motility and abnormal morphology. We also found that the occurrence of *lpgat1* homozygous (*lpgat1*^*−*/*−*^) mutants was far lower than expected. In addition, downregulation of *lpgat1* by morpholino antisense oligonucleotides resulted in severe developmental defects. Lipidomic analysis revealed that selective phospholipid species with stearic acid and docosahexaenoic acid were reduced in homozygous larvae and spermatozoa from heterozygotes. These results suggest that the specific phospholipid molecular species produced by Lpgat1 have an essential role in sperm fertilization and in embryonic development.

## Introduction

In phospholipids, various fatty acids are attached to the *sn*-1 and *sn*-2 positions of the glycerol backbone. Usually, saturated fatty acids (SFAs) such as palmitic acid (C16:0) and stearic acid (C18:0), and monounsaturated fatty acids (MUFAs) such as oleic acid (C18:1), are bound to the *sn*-1 position, while polyunsaturated fatty acids (PUFAs) such as linoleic acid (C18:2), arachidonic acid (C20:4), eicosapentaenoic acid (EPA, C20:5) and docosahexaenoic acid (DHA, C22:6) are bound to the *sn*-2 position. Phospholipids are extremely diverse due to variations in the polar head and the *sn*-1 and *sn*-2 fatty acids; more than 1,000 of them have been identified^1^. Understanding their functions has been the focus of many studies in recent years.

Part of the diversity of phospholipids is determined by lysophospholipid acyltransferases (LPLATs), which incorporate fatty acids into phospholipids^2–7^. LPLATs, which are localized in the endoplasmic reticulum (ER) or mitochondria, recognize both lysophospholipids and acyl-CoAs as substrates, and catalyze the transfer of an acyl chain to the phosphoglycerol backbone of lysophospholipid. To date, 14 LPLAT isozymes are known in humans with different specificities for lysophospholipids and acyl-CoAs. LPLATs are subdivided into two families, the acylglycerophosphate acyltransferase (AGPAT) family and the membrane-bound *O*-acetyl transferase (MBOAT) family^8^.

LPGAT1 is an AGPAT that was first shown to introduce oleic acid into lysophosphatidylglycerol (LPG)^9^ and was later shown to introduce palmitic acid into monoacylglycerol (MAG)^10^. Recently, LPGAT1 knockout was found to protect mice from diet-induced obesity but also to cause liver dysfunction, insulin resistance and nonalcoholic fatty liver disease (NAFLD)^11^.

LPGAT1 is highly conserved in eukaryotes, including *Caenorhabditis elegans* and vertebrates. The LPGAT1s in human and zebrafish are nearly 70% identical at the amino acid level. In this study, we generated Lpgat1 knockout zebrafish using the CRISPR/Cas9 system and analyzed its phenotype. We identified two obvious phenotypes in the mutants: a male fertilization defect and an embryonic developmental defect. In addition, a lipidomic analysis revealed that stearic acid and DHA-containing phospholipids were selectively reduced in Lpgat1-deficient zebrafish larvae and spermatozoa, suggesting that specific phospholipids have important functions during sperm fertilization and embryonic development.

## Results

### Establishment of *lpgat1* zebrafish mutants

**Figure 1:**
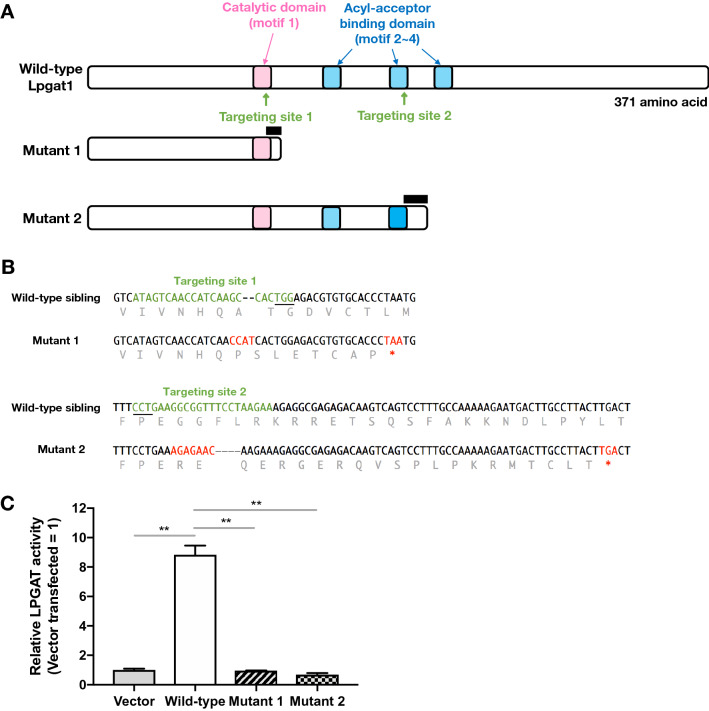
Generation of *lpgat1* mutant zebrafish by CRISPR/Cas9 system. (**A**) Domain structure and targeting sites for disruption of zebrafish Lpgat1 and schematic diagram of mutant Lpgat1 proteins. The active domain (motif 1) and the three acyl-acceptor binding domains (motif 2–4), which are highly conserved in AGPAT family molecules, are indicated by a pink box and blue boxes, respectively. For efficient disruption of Lpgat1 function, two sites near the functional motif were targeted. Both mutants 1 and 2 are predicted to be truncated proteins. The changed amino acid sequences are indicated by black bars. (**B**) Sequence alignment of the mutant alleles with predicted amino acids. Mutated nucleotide sequences and the resulted stop codons are in red. Targeting sites are in green. PAM sequences are underlined. Predicted amino acid sequences are in gray. (**C**) Lysophosphatidylglycerol acyltransferase (LPGAT) activities of wild-type and mutant Lpgat1 proteins. The LPGAT assay was performed using oleoyl lysophosphatidylglycerol (C18:1 LPG), palmitoyl-CoA (C16:0-CoA) and the microsomal fraction from HEK293A cells expressing wild-type or mutant Lpgat1 proteins. Relative LPGAT activities (activity of the microsomal fraction from vector-transfected cells = 1) are shown. The data represents the mean ± S.D. of triplicate measurements. Statistically significant differences are marked with asterisk. **p < 0.01. Two-way ANOVA, Holm’s multiple comparison test was used.

*LPGAT1* genes are conserved in a variety of animal species (Fig. S1A). Zebrafish have a unique ortholog of *LPGAT1* (Gene ID: 561,832) that is about 70% homologous at the amino acid level to human LPGAT1. AGPATs, including LPGAT1s, have four motifs (motifs 1–4) that are required for catalytic activity (Fig. 1A upper and S1B). Using the CRISPR/Cas9 system, we created two zebrafish mutants (mutants 1 and 2) with frameshift mutations near motifs 1 and 3 of *lpgat1*, respectively (Fig. 1A lower two panels and S2). Mutants 1 and 2 were predicted to have truncated Lpgat1 enzymes with complete and partial loss of the active domains, respectively (Fig. 1B). Total RNAs extracted from the mutant larvae were subjected to RT-PCR to amplify the full length cDNAs that cover the ORFs of *lpgat1*. Both mutants 1 and 2 yielded cDNAs of the same length as those obtained from the wild-type larvae, which were found to have the same nucleotide sequence detected in the genome sequence (Fig. S3). When HEK293A cells were transfected with these cDNAs, mutant Lpgat1 proteins were detected as truncated proteins with expected molecular weights on western blotting (Fig. S4). The wild-type enzyme was active whereas the truncated enzymes showed no activity (Fig. 1C), thus confirming that they were inactive.

### Male *lpgat1* heterozygous mutants showed a reproductive defect

F0 founders were derived from the CRISPR/Cas9 injected wild-type embryos. During the establishment of the *lpgat1* mutants from F0 founders by backcrossing them with wild-type zebrafish, we noticed that heterozygous (*lpgat1*^+*/−*^) males showed a reproductive abnormality.

**Figure 2:**
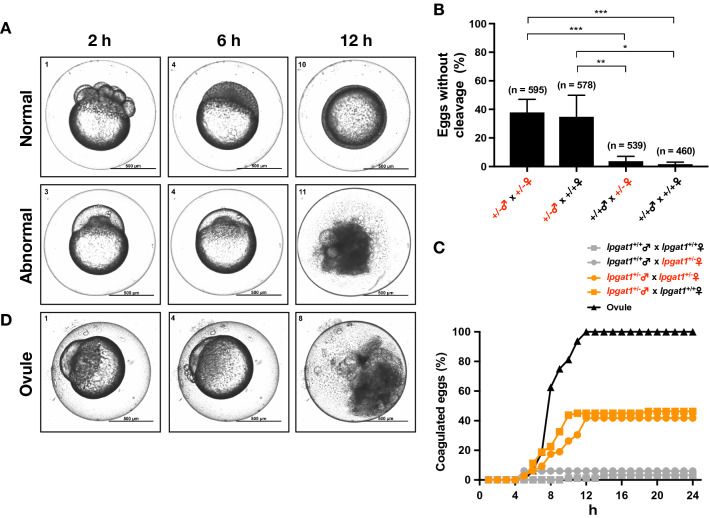
*lpgat1*^+/*−*^ male zebrafish exhibited a reproductive defect. (**A**) Representative images of abnormal eggs resulting from crossing between *lpgat1*^+/*−*^ male and wild-type female. The abnormal eggs showed no signs of cell division and, over time, became coagulated. The results from mutant 1 are shown. The elapsed time after mating is indicated at the top of the panels. Zen Pro software (Zeiss) was used to process the images. Scale bar, 500 µm. (**B**) Percentage of eggs with no sign of cell division at 6 hpf. Results from the crossing between the indicated genotypes are shown. For mutants 1 and 2, four genotype combinations of crosses (*lpgat1*^+/+^ males/*lpgat1*^+/+^ females, *lpgat1*^+/*−*^ males/*lpgat1*^+/+^ females, *lpgat1*^+/+^ males/*lpgat1*^+/*−*^ females and *lpgat1*^+/*−*^ males/*lpgat1*^+/*−*^ females) were performed for four times each and the results of the same genotype combination were combined. The numbers of eggs analyzed are indicated on the top of each bar. Error bars are S.D. Statistically significant differences are marked with asterisk. *p < 0.05; **p < 0.01, ***p < 0.001. Two-way ANOVA, Holm’s multiple comparison test was used. (**C**) Percentage of coagulated eggs overtime after mating. Results from the crossing between the indicated genotypes are shown. Crossing were performed for four times each. Representative data using mutant 1 are shown. Eighty eggs were buried in an agarose gel and evaluated based on the images by time-lapse observation. The result from ovules (unfertilized eggs) is also shown. (**D**) Representative images of ovules (unfertilized eggs) squeezed out of a wild-type female over time. Note that they showed no signs of cell division and, over time, became coagulated as in (**A, lower**). The elapsed time after the eggs were squeezed out is indicated at the top of the panel. Scale bar, 500 µm.

Time-lapse observations of the eggs obtained from crosses between heterozygous males and wild-type females immediately after mating revealed that these eggs did not show any sign of cleavage at the initial stage of development (2–6 h post fertilization (hpf)) (Fig. 2A lower). At 6 hpf, about 40% of the eggs had not cleaved and this was observed only in crosses with the male heterozygotes. (Fig. 2B). With time, the contents of these abnormal eggs became coagulated, and at 12 hpf almost all of them showed a coagulation phenotype, a typical phenotype of eggs with developmental abnormalities, resulting in a nearly 40% abnormal eggs at 12 hpf (Fig. 2C and Supplemental Movie). The rest of the eggs developed normally and did not show any defects during the early developmental stages (~ 24 hpf) (Fig. 2A upper and 2C). Interestingly, ovules squeezed out of wild-type females normally showed a phenotype similar to the coagulation phenotype (Fig. 2D), suggesting that unfertilized eggs were more likely to be present in the crosses between heterozygous males and wild-type females. In both mutants1 and 2, ovule-like eggs were detected only when the male was a heterozygote and were not detected when wild-type males were crossed with heterozygous females. (Fig. 2B,C).

**Figure 3:**
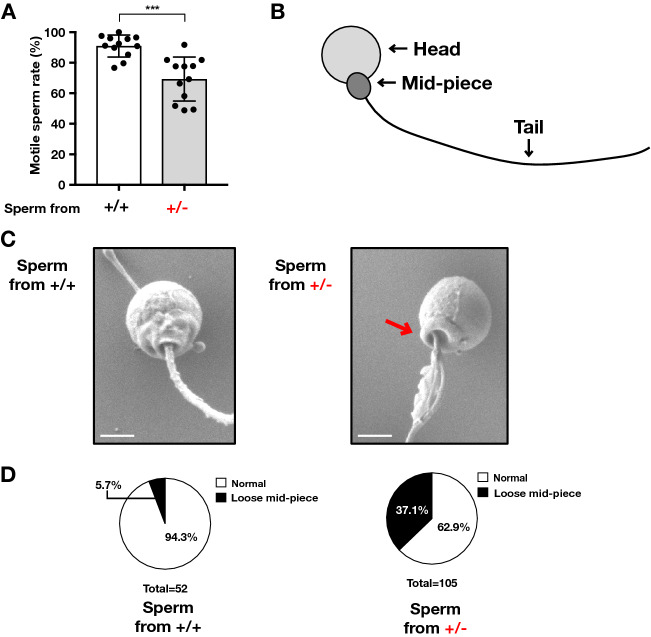
*lpgat1* heterozygotes produce some sperm with less motility and abnormal morphology. (**A**) Percentage of sperm with motility from *lpgat1*^+/+^ and *lpgat1*^+/*−*^. Semen was collected from anesthetized zebrafish by abdominal compression. Sperm motility was measured by Computer Assisted Semen Analysis, CASA. Each point represents the results of different individual zebrafish (n = 12 for both *lpgat1*^+/+^ and *lpgat1*^+/*−*^, The data were combined from mutant 1 (n = 7) and 2 (n = 5)). Error bars are S.D. Statistically significant differences are marked with asterisks. *p < 0.05; **p < 0.01. Unpaired, two-tailed *t*-test was used. (**B**) Schematic diagram of zebrafish sperm. Zebrafish sperm consists of a head, a mid-piece, and a tail. Unlike sperm from mammals, sperm from zebrafish have no acrosome. The head contains the nucleus, and the midpiece is enriched in mitochondria. (**C**) SEM analyses of sperm from *lpgat1*^+/+^ and *lpgat1*^+/*−*^ zebrafish. Representative images from mutant 1 were shown. Note that sperm from *lpgat1*^+/*−*^ showed abnormal morphology with loose mid-piece indicated by red arrows. Scale bar, 1 µm. (**D**) Percentage of sperm with abnormal morphology in SEM analyses (**C**). Sperm from *lpgat1*^+/+^; n = 52, from *lpgat1*^+/*−*^ (mutant 1); n = 105.

Microscopic analyses revealed that the sperm from heterozygous males were less motile than those from wild-type males (Fig. 3A). In addition, scanning electron microscopy (SEM) images of sperm from a heterozygote revealed abnormalities in the mid-piece, the structure that links the neck to the head (Fig. 3B–D). These results demonstrated that *lpgat1* has a critical role in sperm motility and structure. They also indicate that the reduced motility and abnormal morphology of the sperm underlie the reproductive abnormalities of heterozygous males.

We also determined the genotypes of the embryos that survived and grew from the crosses between heterozygous males and wild-type females. Interestingly, wild-type and heterozygotes were found to be present in the same proportions (48.1% and 51.9%) in the surviving embryos as was observed with embryos from the crosses between wild-type males and heterozygous females (Fig. S5 and S6), suggesting that sperm with either the *lpgat1* (−) or *lpgat1* ( +) allele have comparable abnormalities.

### *lpgat1* homozygous mutants are developmentally abnormal and did not grow to adulthood

**Figure 4:**
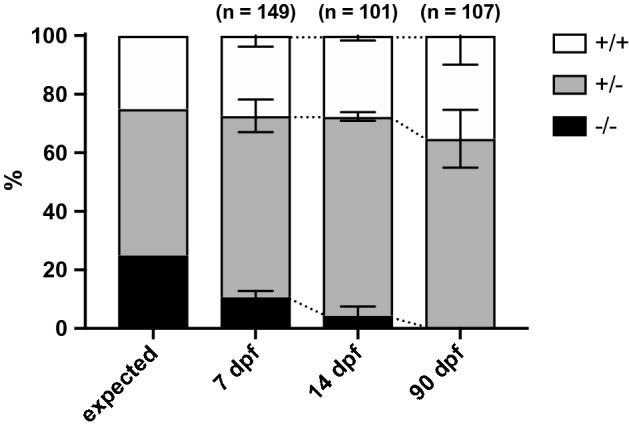
*lpgat1* homozygous mutants were not reproduced with the expected Mendelian ratio. Genotypes of offspring from crossing between heterozygotes (*lpgat1*^+/−^). The genotypes of littermate zebrafish obtained by crossing *lpgat1*^+/−^ males and females were determined at each time point. Data at 7, 14, and 90 dpf were obtained from 4, 3, and 7 independent crosses, respectively. The data of mutants 1 and 2 were combined. The numbers of larvae analyzed are indicated on the top of each bar. Error bars are S.D.

In an effort to produce homozygous *lpgat1* mutants (*lpgat1*^*−*/*−*^) by crosses between heterozygotes (*lpgat1*^+/*−*^), we found that homozygous mutants were produced at less than the expected Mendelian ratio and that the ratio continued to decline with time (Fig. 4). Some of the homozygous mutants survived to 14 dpf (days post fertilization). Although not statistically significant, only one homozygous of 24 juveniles produced from crosses between heterozygotes survived to 30 dpf. By contrast, the proportions of heterozygotes and wild-type juveniles were almost as expected (wild-type: heterozygotes = 1: 2) (Fig. 4). However, we did not observe any morphological differences among homozygous mutants, heterozygous mutants and wild-type juveniles (data not shown). The cause of abnormalities and death was unclear because about 25% of wild-type juveniles die within two weeks of fertilization. In addition, approximately 40% of eggs obtained from crosses between heterozygous males and females do not develop due to fertilization abnormalities of the heterozygous males (Fig. 2).

### Downregulation of *lpgat1* causes developmental abnormalities in the embryos

**Figure 5:**
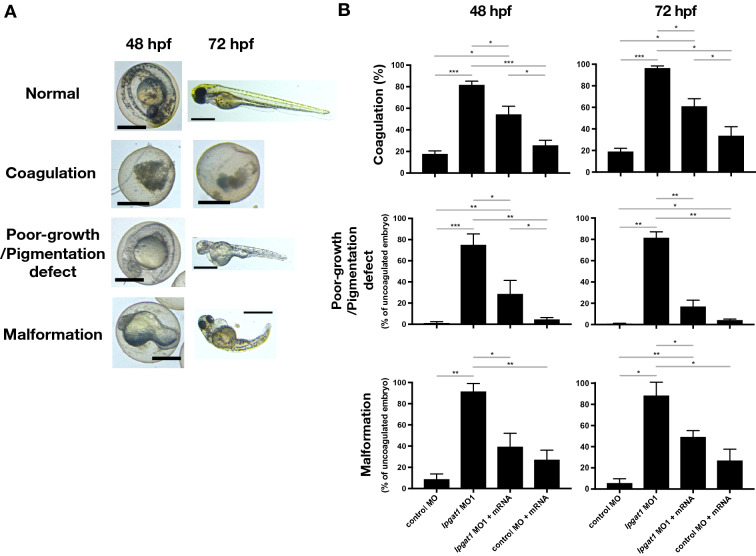
Downregulation of *lpgat1* induced various embryonic developmental defects using morpholino oligonucleotides (MO). Each of embryos injected with MOs were observed overtime by time-lapse imaging. (**A**) The representative images of the normal embryos and larva, coagulated eggs, embryos and larva with poor-growth/pigmentation defect, and malformed embryo (with bent body axis, short body length and/or edema). LAS software (Ver. 4.12, Leica) was used to process the images. hpf; hours post fertilization. Scale bar, 500 µm. (**B**) Upper graphs, percentage of coagulated eggs after injection of morpholino antisense oligo (MO) specific to *lpgat1* (MO1) or control MO (cMO) in combination with *lpgat1* mRNA. MO1 is a splicing inhibitor, which bind to the exon–intron junction of immature *lpgat1* mRNA and inhibit the splicing of pre-mRNA. Middle graphs, percentage of pigmentation defect and bottom graphs, malformation in uncoagulated embryos. Basically, the same results were obtained as shown in Fig. S9B using morpholino oligonucleotides 2 (MO2). The means of the data from three independent experiments for each group with n = 200 in total are shown. Error bars are S.D. Statistically significant differences are marked with asterisk. *p < 0.05; **p < 0.01, ***p < 0.001. Two-way ANOVA, Holm’s multiple comparison test was used.

To clarify the role of *lpgat1* in development, we suppressed the expression of *lpgat1* in embryos by injecting morpholine oligonucleotides (MO) that specifically inhibit splicing of *lpgat1* pre-mRNA. Compared with control MO-injected embryos, embryos injected with the MOs showed a coagulated phenotype starting at 48 hpf (Fig. 5, S8, S9A and B), which is a well-known indication that embryo development has stopped. At this time point, about 80% of the embryos showed a coagulated phenotype, but we also observed embryos that showed a poor-growth/pigmentation defect and malformation with edema were observed in addition (Fig. 5, S8, S9A and B). At 72 hpf, most (> 90%) of the embryos had the coagulated phenotype (Fig. 5B and S9B). The remaining embryos either did not hatch or hatched late with severe malformations, including a bent body axis, short body length (Fig. 5 and S9B), no heart beating (Fig. S9C). These abnormalities were significantly suppressed by simultaneous injection of zebrafish *lpgat1* mRNA (Fig. 5, S8 and S9). Of note, at 72 hpf, morphologically normal hatched larvae were present in the mRNA co-injected groups. These analyses revealed that Lpgat1 and its products have critical roles in various stages of late embryonic development of zebrafish.

### Lipidomic analyses of the *lpgat1* mutants

**Figure 6:**
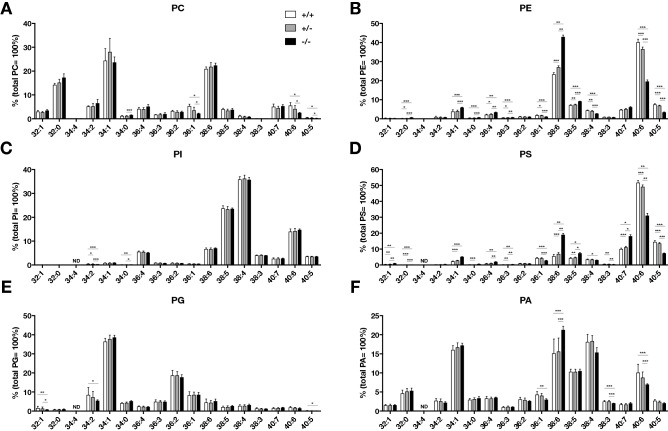
Lipidomic analyses of *lpgat1* mutant zebrafish. Lipids were collected at 7 days post fertilization from a single zebrafish larva obtained from crossing an *lpgat1*^+/−^ male with an *lpgat1*^+/−^ female and analyzed for phosphatidylcholine (PC, **A**), phosphatidylethanolamine (PE, **B**), phosphatidylinositol (PI, **C**), phosphatidylserine (PS, **D**), phosphatidylglycerol (PG, **E**), and phosphatidic acid (PA, **F**) by LC–MS/MS. Genotypes of each larva were determined by PCR after a lipid sample was collected. The fish were obtained from three independent crosses. The numbers of samples were n = 18 for *lpgat1*^+/+^, n = 19 for *lpgat1*^+/−^ and n = 7 for *lpgat1*^−/−^. Error bars are S.D. Statistically significant differences are marked with asterisks. *p < 0.05; **p < 0.01; ***p < 0.001. Two-way ANOVA, Bonferroni’s multiple comparison test was used. For molecular species with low abundance, the relative abundance of each molecular species is shown in Supplementary Fig. S9.

An LC–MS/MS analysis of the lipids of *lpgat1* mutant larvae (homozygous mutants and heterozygotes) showed that the loss of Lpgat1 reduced the levels of multiple phospholipids (Fig. 6 and S10) and diacylglycerols (DGs) (Fig. S11). Compared to wild-type larvae, *lpgat1* mutant larvae had significantly lower levels of phosphatidylethanolamine (PE) and phosphatidylserine (PS) species, and slightly different levels of phosphatidylcholine (PC) and phosphatidic acid (PA) species. By contrast, we did not observe significant changes in the levels of phosphatidylglycerol (PG) and phosphatidylinositol (PI) species (Fig. 6 and S10). Interestingly, a decrease in the C40:6 species and an increase in the C38:6 species were commonly observed for PE, PS, PC, and PA in varying degrees. MS/MS analysis revealed that C40:6 species were composed of stearic acid (C18:0) and docosahexaenoic acid (DHA, C22:6), while C38:6 species were composed of palmitic acid (C16:0) and DHA (data not shown). These changes were most obvious in PE and PS. As for DGs, C38:6 species was slightly decreased in homozygous larvae (Fig. S11). It should be stressed that the changes in the lipid species were greater in the homozygous mutants, but essentially the same changes were observed in heterozygotes, although to a lesser extent. The total amount of all molecular species for PC, PE, PI, PS, PG or PA was not significantly different among them (Fig. S12).

**Figure 7:**
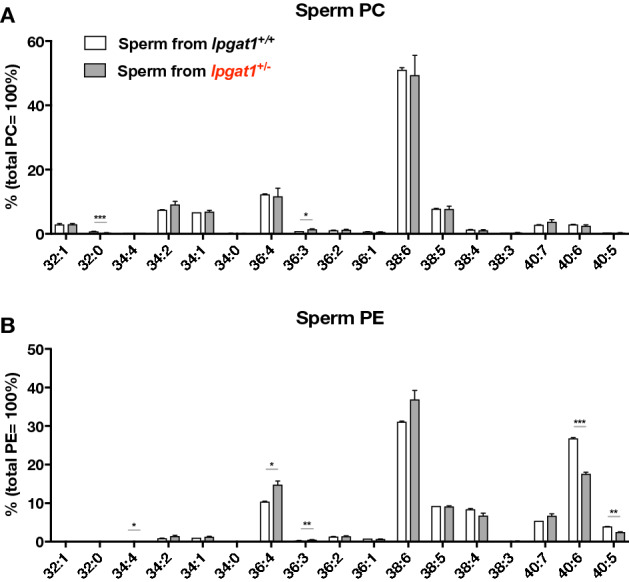
Phosphatidylcholine (PC) and phosphatidylethanolamine (PE) species in sperm from *lpgat1*^+/−^ zebrafish. PC (**A**) and PE (**B**) species composition of zebrafish sperm was analyzed by LC–MS/MS. Semen was collected from an anesthetized zebrafish by abdominal compression. Sperm pellets were prepared from semen collected from one zebrafish and lipids were recovered. The numbers of sperm samples were n = 4 for *lpgat1*^+/+^ and n = 4 for *lpgat1*^+/−^. Error bars are S.D. Statistically significant differences are marked with asterisks. *p < 0.05; **p < 0.01; ***p < 0.001. Unpaired, two-tailed t test was used. For molecular species with low abundance, the relative abundance of each molecular species is shown in Supplementary Fig. S12.

The abundances of PE species in the sperm from wild-type and heterozygotes were similar. Of note, an increase in C40:6 (C18:0–C22:6) species and a decrease in C38:6 (C16:0–C22:6) species were observed in PE species (Fig. 7B and S13B) but not in PC species (Fig. 7A and S13A). The levels of PS, PG and PA molecular species in sperm (data not shown) were too low to measure accurately.

## Discussion

The present results show that Lpgat1 mutant zebrafish have two obvious phenotypes associated with male reproduction and one associated with embryonic development: abnormal male reproduction (Fig. 2A,B), abnormal motility and morphology of some spermatozoa (Fig. 3), and decreased ratio of homozygous mutants in the developmental stages (Fig. 4).

The abnormal fertility of spermatozoa from heterozygotes appears to be responsible for the abnormal male reproduction. Zebrafish eggs have a single micropyle that is wide enough to allow only a single sperm to enter^12^. The lower motility (Fig. 3A) and abnormal morphology (Fig. 3B–D) of some sperm from heterozygotes likely impeded their passage through the micropyle. Despite the abnormality of many of the spermatozoa from heterozygotes, almost equal numbers of *lpgat1* heterozygotes and wild-type embryos were obtained from a cross between heterozygous males and wild type females (Fig. S5 and S6). We speculate that not only spermatozoa with the *lpgat1* (–) allele but also those with the *lpgat1* ( +) allele have abnormalities to a similar degree. In other words, the abnormality of spermatozoa from heterozygous males is independent of their genotype. In general, during spermatozoon formation, spermatogonia undergo two cell divisions, *i.e.*, meiosis-1 and -2. In meiosis-1, paternal and maternal chromosomes segregate to form diploid cells, and in meiosis-2, each chromosome further segregates to form haploid spermatozoa. We confirmed that *lpgat1* mRNA is highly expressed in zebrafish testes, as judged by in situ hybridization (Fig. S7). Our present data indicate that *lpgat1* is expressed in spermatogonia and spermatocytes before meiosis-2 and has a crucial role in spermatozoon formation. The *Lpgat1* KO mice were reported to be resistant to obesity, which the authors attributed to abnormal mitochondrial function^11^. Abnormal mitochondrial function might reduce ATP production, which could explain the reduced motility of sperm from heterozygotes.

In crosses between heterozygotes, the number of homozygous mutants decreased rapidly with time (Fig. 4), suggesting that homozygous mutants died at different stages of development. Thus, we were unable to obtain adult of homozygous mutants under our breeding conditions. Because we obtained a limited number of homozygous mutants in this study, we could not determine the cause of lethality in juveniles after 7 dpf. Recently, another group reported that *Lpgat1* KO mice had small body size immediately after birth^11^. However, unlike the homozygous zebrafish mutants, KO mice were born according to Mendelian rules and the KO was not embryonic lethal. The cause of the death in zebrafish is currently unknown and remains to be solved. Interestingly, however, *Lpgat1* KO mice, which are now being analyzed in our laboratory, show a sudden death phenotype, with about half of the mice dead by 8 weeks of age (data not shown).

On the other hand, analysis of *lpgat1* knockdown using morpholino oligonucleotides (MOs) revealed developmental abnormalities in the late stage of embryonic development (Fig. 5, S8 and S9). In fact, several of typical developmental abnormalities were observed at 48 and 72 hpf, including coagulation, poor-growth/pigmentation defect, malformation, and hatching defect. These phenotypes were partially rescued by simultaneously injecting the embryos with *lpgat1* mRNA, which excludes the possibility that the phenotypes are off-target effects of MOs, which is often problematic in functional studies using MOs^13,14^.

The *lpgat1* mutants showed changes in various phospholipid classes (Fig. 6). One of them was PE which has been reported to be closely associated with mitochondrial function. The major pathway for PE production in mitochondria is PS decarboxylation, and deficiency of PS decarboxylase resulted in embryonic developmental abnormalities and embryonic lethality due to reduced PE levels and abnormal mitochondrial function in mice^15^. Thus, alterations in the PE molecular species in *lpgat1* mutant zebrafish may be involved in the mitochondrial abnormalities, which is our next challenge.

Among the changes in different phospholipid classes, a decreased abundance of C40:6 species was commonly observed in the PC, PE, PS and PA phospholipid classes. The MS/MS spectra indicated that these phospholipid species contain stearic acid (C18:0) and DHA (C22:6). In addition, we found an increase in the number of PC, PE and PS molecular species with shorter acyl chains (Fig. 6A,B,D). The MS/MS analysis showed that these phospholipid species contained palmitic acid (C16:0) (data not shown). Stearic acid, but not palmitic acid, was recently found to be critical in modulating mitochondria morphology and function^16^, raising the possibility that Lpgat1 exerts its effect by producing stearic acid-containing phospholipids.

Lipids are critical components in spermatogenesis. For example, albumin-bound lipids such as lysophospholipids are critical for inducing mouse spermatogenesis even under organ culture conditions^17^. Phospholipids containing PUFAs, especially DHA, are important for the formation and function of sperm^18–20^. On the other hand, phospholipids containing SFAs may also be necessary, as palmitic acid (C16:0)-containing phospholipids such as tetrapalmitoylcardiolipin are present in specific stages in spermatogenesis in both mice and humans^21^.

Interestingly, changes in phospholipid molecular species similar to the ones we detected in Lpgat1 mutant zebrafish were detected in seminal plasma from infertile human males and the changes were reported to be correlated with sperm motility^22^. The authors showed a significant decrease in the PE level and an increase in the PS level, as well as an increase in palmitic acid-containing species and a decrease in stearic acid-containing species levels. They also suggested that specific PE species, e.g. DHA-containing PE, decreased, suggesting that the loss of *lpgat1* may alter the composition of specific phospholipid species, which somehow affects the morphology and motility of sperm.

In summary, we have shown that zebrafish lacking the specific phospholipid-metabolizing enzyme Lpgat1 showed abnormal fertilization and growth phenotypes. Our next goal is to identify the phospholipid molecular species involved in each phenotype and to elucidate the specific functions of these phospholipid species.

## Materials and methods

### Zebrafish

A wild-type AB zebrafish strain was obtained from Zebrafish International Resource Center (University of Oregon, OR, USA). Fishes were maintained at 28 °C under the controlled 13.5 h light/10.5 h dark cycle. Embryos were obtained from natural spawning and kept at 28 °C in E2 medium (15 mM NaCl, 0.5 mM KCl, 1 mM MgSO_4_, 0.15 mM KH_2_PO_4_, 0.05 mM Na_2_HPO_4_, 1 mM CaCl_2_, 0.7 mM NaHCO_3_) supplemented with 0.1 mg/L methylene blue. All animal experiments were performed in accordance with institutional and national guidelines and regulations. The study was carried out in compliance with the ARRIVE guidelines and approved by the Animal Care and Use Committee of Graduate school of Pharmaceutical Sciences of the University of Tokyo (Approval Identification Number: 3–34).

### Generation of *lpgat1* mutant fish by CRISPR/Cas9 system

*lpgat1* mutant fish were generated as previously reported^23^ with modifications. crRNAs targeting *lpgat1* were designed using online software CRISPRdirect^24^ and listed below. The individual synthetic crRNAs, synthetic tracrRNA, and recombinant Cas9 protein were obtained from FASMAC (Kanagawa, Japan). crRNA (50 pg) and tracrRNA (160 pg) with Cas9 Protein (400 pg) were co-injected into 1-cell stage zebrafish embryos (F0 founder) derived from wild-type fish. The sequences of the two crRNAs used are as follows (See also Fig. 1 and S2A).

crRNA 1 (for targeting site 1): AUAGUCAACCAUCAAGCCACUGG

crRNA 2 (for targeting site 2): UUUCUUAGGAAACCGCCUUCAGG.

The mutants were analyzed using the mainly F6 generations, which were generated by crossing the CRISPR/Cas9-injected F0 founder with the wild type six times.

### Lysophospholipid acyltransferase assay

cDNA encoding wild-type zebrafish Lpgat1 was cloned by RT-PCR amplification using the following oligonucleotide primers and a zebrafish 7 dpf larva cDNA as a template.

Fwd: GGTACCATGGCTCCCCATCTGGACGT

Rev: TCAGAAGAGCCAGAAGTAGA.

The Amplified PCR products were cloned into an expression vector pCAGGS and sequenced. Similarly, cDNAs encoding mutant1 and 2 Lpgat1 were amplified by RT-PCR and inserted into a pCAGGS vector. Transfection and enzymatic assay were performed as describe previously^25^. In brief, HEK293A cells were transfected with cDNAs encoding wild-type and mutant Lpgat1 proteins. After 24 h, the cells were harvested and suspended in TSC buffer (20 mM Tris–HCl, pH 7.4, 300 mM sucrose and cOmplete™ Protease Inhibitor Cocktail (Roche, Mannheim, Germany)) and were sonicated. After centrifugation for 10 min at 800 × g the supernatants were collected and centrifuged at 100,000 × g for 1 h. The resulting pellet was resuspended in TSE buffer (20 mM Tris–HCl, pH 7.4, 300 mM sucrose and 1 mM EDTA). Protein concentrations were measured with a BCA protein assay kit (Thermo Fisher Scientific). Lysophospholipid acyltransferase activity was measured by the following procedure. Reaction mixtures contained 110 mM Tris–HCl (pH 7.4), 150 mM sucrose, 0.5 mM EDTA, 10 μM oleoyl-LPG as an acyl acceptor, 2 μM palmitoyl-CoA as an acyl donor, 0.015% Tween-20 and 0.1 μg of membrane protein as an enzyme source. After incubation at 37 °C for 10 min, reactions were stopped by the addition of chloroform: methanol (1:2, v/v). Dilauroyl (di12:0) PG was added as an internal standard and total lipids were extracted by the Bligh-Dyer method, and subsequently, the assay products (palmitoyl-oleoyl-PG) were measured by LC–MS/MS.

### Western Blotting

Membrane fractions of HEK293A cells transfected with cDNA encoding wild-type or mutant Lpgat1 proteins were dissolved in SDS-PAGE sample buffer (50 mM Tris–HCl (pH 7.4), 50 mM dithiothreitol, 150 mM NaCl, 1% (v/v) TritonX-100, 0.5% (w/v) sodium deoxycholate, 0.1% SDS and 20 mM EDTA). Samples containing 1 µg membrane proteins were separated by 15% SDS-PAGE and transferred to polyvinylidene fluoride (PVDF) membranes (Millipore). After incubation with 5% (w/v) skimmed milk in TTBS buffer (10 mM Tris–HCl (pH 7.4), 150 mM NaCl, 0.05% (w/v) Tween 20) for 1 h at room temperature, the PVDF membranes were further incubated with primary and then secondary antibodies. Primary antibodies used in this study were mouse anti-DYKDDDDK (FLAG) monoclonal antibody (Clone 2H8, Trans Genic Inc., KO602-L, lot TG071014, 1/4,000 dilution) and anti-Calnexin rabbit polyclonal antibody (Abcam, ab22595, lot GR3298210-1, 1/4,000 dilution). Secondary antibodies that were conjugated with horseradish peroxidase (HRP) were Sheep anti-mouse IgG HRP-Linked F(ab’)2 Fragment (GE Healthcare, NA9310, lot 16,997,947) and Donkey anti-rabbit IgG HRP-Linked F(ab’)2 Fragment (GE Healthcare, NA9340, lot 17,065,618). The PVDF membranes were soaked with a luminol reagent (100 mM Tris–HCl (pH 8.5), 50 mg/ml Luminol Sodium Salt HG (FujiFilm Wako Pure Chemical), 0.2 mM *p*-Coumaric acid and 0.03% (v/v) of H_2_O_2_). A chemiluminescence image was acquired with ImageQuant LAS 4000 system (GE Healthcare).

### Time-lapse imaging

Time-lapse bright-field images were acquired with an Axio Observer Z1 microscope (Zeiss, Oberkochen, Germany). Embryos were embedded in 1% low-melting-point agarose dissolved in E2 medium in a glass bottom 12-well plate (Matsunami, Osaka, Japan). Zen Pro software (Zeiss) was used to process the images.

### Heteroduplex mobility assay

To prepare the genomic DNA, embryos were incubated in 90 µL of 50 mM NaOH at 98 °C for 10 min. Then, 10 µL of 1 M Tris–HCl (pH 8.0) was added. Genomic fragments at the target sites were amplified by PCR. The PCR amplicons were electrophoresed on a non-denaturing polyacrylamide gel containing 15% acrylamide/bisacrylamide (29:1, w/w), 25 mM tris(hydroxymethyl)aminomethane, 120 mM glycine, ammonium persulfate, and *N*,*N*,*N*’,*N*’-tetramethyl ethylenediamine. The gel polymerized at room temperature. After 70 min of constant current electrophoresis at 30 mA, the gel was stained with ethidium bromide solution (500 ng/ml) for 10 min, and data was acquired using a LAS4000 (GE Healthcare, IL, USA). The locus-specific primers were listed below:

Mutant 1 Fwd: ACAGAATGGGGTGATGATGTGA

Mutant 1 Rev: TAGCAGACTTACCGTGCCCTT

Mutant 2 Fwd: AGAAAAGCAGCTCGTGTACCT

Mutant 2 Rev: AGGACTGACTTGTCTCTCGCC

### Sperm motility analysis

Zebrafish spermatozoa were expelled by gently squeezing the sides of the lower abdomen of the male fish with filter tweezers, and was collected in Hank’s buffer (137 mM NaCl, 5.4 mM KCl, 0.25 mM Na_2_HPO_4_, 1.3 mM CaCl_2_, 1 mM MgSO_4_, and 4.2 mM NaHCO_3_). Spermatozoa were inactive in Hank’s buffer, but were activated by being diluted 10 times with water. Sperm motility was analyzed by Computer Assisted Semen Analysis, CASA (SMAS; Detect, Tokyo Japan). Spermatozoa were immediately filled into SMAS chamber (DP041125; Detect) after dilution with water and motility was measured two times of 1 s. The average value of the two measurements was used as the result.

### SEM analysis

Zebrafish spermatozoa were fixed using 2.5% glutaraldehyde and suspended in 2% paraformaldehyde dissolved in 20 mM HEPES (pH 7.2), then dropped onto a round glass slide with poly-L-lysine coating. After 24 h incubation at 4 °C, slide was washed two times with 20 mM HEPES (pH 7.2). After the surface was treated with electric conduction, the sperm was observed under a scanning electron microscope (JSM-6701F; JEOL, Tokyo, Japan).

### RNA in situ hybridization

Testes were removed by dissection and then fixed overnight with Bouin’s fixative (Wako, Osaka, Japan) at 4 °C and then embedded in O.C.T. compound (Sakura Finetek, Tokyo, Japan). The tissues were cryosectioned at 10 µm and plated on MAS-coated glass slides (Matsunami Glass, Osaka, Japan) and processed for RNA in situ detection using the RNAscope 2.0 High Definition RED kit according to the instructions of the manufacturer (ACDBio, MN, USA). The negative probe detects the *E. coli dapB* gene, which encodes dihydrodipicolinate reductase.

### Morpholino antisense oligo (MO) and mRNA injection

MOs designed to bind to exon–intron junctions of pre-mRNAs are listed below. MOs were obtained from Gene Tools, LLC (OR, USA). Control MO (cMO) targets the human beta-globin intron mutation and has little effect on gene expression in zebrafish.

MOs were dissolved in water with 0.02% phenol red. mRNA was synthesized in vitro*.* Template DNA was amplified by PCR from *lpgat1* cording plasmid vector. Then mRNA was synthesized (mMESSAGE mMACHINE T7 kit; Invitrogen, MA, USA), poly(A) was added (Poly(A) tailing kit; Invitrogen), and purified (MEGAclear kit; Invitrogen). Two nl of 80 µM MO, or 80 µM MO and 100 ng/µl mRNA reagents were injected per embryo performed by manual injector and had been completed until 2 cell stage.

MO 1: AGGGAAATGTTGTTCTTACCTGTGT

MO 2: AAAAAATAGCAGACTTACCGTGCCC

Each of embryos injected with MOs were observed by time-lapse imaging for 72 h.

### LC–MS/MS sample preparation

A zebrafish larva or sperm was placed in a 1.5-ml siliconized sample tube. Sperm collected in Hank’s buffer was centrifuged to make a pellet. The supernatant was removed. Then 70 µl of isopropanol, containing 1 µM dilauroyl (di12:0) phosphatidylcholine (PC), 1 µM di12:0 phosphatidylethanolamine (PE), 1 µM di8:0 phosphatidylinositol (PI), 1 µM di12:0 phosphatidylserine (PS), 100 nM phosphatidylglycerol (PG), and 100 nM phosphatidic acid (PA) for an internal standard, was added to the tube. The obtained mixture was sonicated for 10 min in an Ultrasonic bath (AS ONE, Osaka, Japan). After a centrifugation at 21,500 × *g* for 5 min at 4 °C, the supernatant was passed through a filter (0.2 µm pore size, 4 mm inner diameter; YMC, Kyoto, Japan), and 10 µl of the filtrate was analyzed by LC–MS/MS.

### LC–MS/MS analysis

PLs were measured as previously reported^26^ with some modifications. Samples were resolved by the ultra-performance liquid chromatography (UPLC) on a reverse-phase column (L-column 3 C18, particle size 2 µm, inner diameter 2.0 mm, length 100 mm; CERI, Tokyo, Japan) at 45 °C, and detected by ESI tandem quadrupole MS (Xevo TQ-XS; Waters, MA, USA). The flow rate was 0.3 ml/min in a binary gradient system using a mobile phase A (acetonitrile/water (3:2, vol/vol) containing 10 mM ammonium formate) and a mobile phase B (isopropanol/acetonitrile (9:1, vol/vol) containing 10 mM ammonium formate). The gradient steps were as follows: 0 min, 5% B; 0–2 min, 5% B; 2–26.4 min, linear gradient to 100% B; 26.4–36 min, 100% B; 36–36.1 min, linear gradient to 5% B; 36.1–40 min, 5% B. The ESI parameters were as follows: capillary voltage, 2.5 kV; sampling cone voltage, 30 V; desolvation temperature, 400 °C. Each PC species was detected by multiple reaction monitoring (MRM) mode by selecting the *m/z* ([M + H]^+^) of specific PC species at MS1 and the *m/z* 184.1 at MS2 and using the collision energy 30 eV. Each PE or PS species was detected by MRM mode by selecting the *m/z* ([M + H]^+^) of specific PE or PS species at MS1 and the *m/z* ([M-OH]^+^) of specific diacylglycerol (DG) fragments at MS2 and using the collision energy 26 eV of 30 eV, respectively. Each PI, PG, or PA was detected by MRM mode by selecting the *m/z* ([M + NH_4_]^+^) of specific PI, PG, or PA species at MS1 and the *m/z* ([M-OH]^+^) of specific DG fragments at MS2 and using the collision energy 19 eV. Each DG was detected by MRM mode by selecting the *m/z* ([M + NH_4_]^+^) of specific DG species at MS1 and the *m/z* ([M-OH]^+^) of specific monoacylglycerol fragments at MS2 and using the collision energy 10 eV. Data analysis and quantification were performed using MassLynx software and TargetLynx software (Waters).

### Statistical analysis

Unpaired *t-*tests were used to compare two groups. Multiple comparisons were performed with Bonferroni’s or Holm’s multiple comparison tests depending on the combinations of comparisons, after two-way ANOVA. All analyses were done with GraphPad Prism 7 for Mac OS software (GraphPad Software, Inc., CA, USA) and Microsoft Excel software (Microsoft, Redmond, WA, USA).

## Supplementary Information

Supplementary Video 1.

Supplementary Information 1.

## Data Availability

The datasets used and/or analyzed during the current study available from the corresponding author on reasonable request.

## References

[1] Tsugawa H (2020). A lipidome atlas in MS-DIAL 4. Nat Biotechnol.

[2] Shindou H (2017). Docosahexaenoic acid preserves visual function by maintaining correct disc morphology in retinal photoreceptor cells. J. Biol. Chem..

[3] Nakanishi, H. *et al.* Cloning and characterization of mouse lung-type acyl-CoA:lysophosphatidylcholine acyltransferase 1 (LPCAT1). Expression in alveolar type II cells and possible involvement in surfactant production. *J. Biol. Chem.* **281**, 20140–20147. 10.1074/jbc.M600225200 (2006). 10.1074/jbc.M600225200 16704971

[4] Shindou, H. *et al.* A single enzyme catalyzes both platelet-activating factor production and membrane biogenesis of inflammatory cells. Cloning and characterization of acetyl-CoA:LYSO-PAF acetyltransferase. *J. Biol. Chem.* **282**, 6532–6539. 10.1074/jbc.M609641200 (2007). 10.1074/jbc.M609641200 17182612

[5] Hashidate-Yoshida, T. *et al.* Fatty acid remodeling by LPCAT3 enriches arachidonate in phospholipid membranes and regulates triglyceride transport. *Elife* **4**. 10.7554/eLife.06328 (2015). 10.7554/eLife.06328 PMC4436788 25898003

[6] Lee HC (2012). LPIAT1 regulates arachidonic acid content in phosphatidylinositol and is required for cortical lamination in mice. Mol. Biol. Cell..

[7] Tanaka Y (2021). LPIAT1/MBOAT7 depletion increases triglyceride synthesis fueled by high phosphatidylinositol turnover. Gut.

[8] Hishikawa D (2008). Discovery of a lysophospholipid acyltransferase family essential for membrane asymmetry and diversity. Proc. Natl. Acad. Sci. U S A.

[9] Yang Y, Cao J, Shi Y (2004). Identification and characterization of a gene encoding human LPGAT1, an endoplasmic reticulum-associated lysophosphatidylglycerol acyltransferase. J. Biol. Chem..

[10] Hiramine Y, Emoto H, Takasuga S, Hiramatsu R (2010). Novel acyl-coenzyme A:monoacylglycerol acyltransferase plays an important role in hepatic triacylglycerol secretion. J. Lipid. Res..

[11] Zhang X (2019). Defective Phosphatidylglycerol Remodeling Causes Hepatopathy, Linking Mitochondrial Dysfunction to Hepatosteatosis. Cell Mol. Gastroenterol. Hepatol..

[12] Wolenski JS, Hart NH (1987). Scanning electron microscope studies of sperm incorporation into the zebrafish (Brachydanio) egg. J. Exp. Zool..

[13] Kok FO (2015). Reverse genetic screening reveals poor correlation between morpholino-induced and mutant phenotypes in zebrafish. Dev. Cell..

[14] Gerety SS, Wilkinson DG (2011). Morpholino artifacts provide pitfalls and reveal a novel role for pro-apoptotic genes in hindbrain boundary development. Dev. Biol..

[15] Steenbergen R (2005). Disruption of the phosphatidylserine decarboxylase gene in mice causes embryonic lethality and mitochondrial defects. J. Biol. Chem..

[16] Senyilmaz-Tiebe D (2018). Dietary stearic acid regulates mitochondria in vivo in humans. Nat. Commun..

[17] Sanjo H (2020). Antioxidant vitamins and lysophospholipids are critical for inducing mouse spermatogenesis under organ culture conditions. FASEB J..

[18] Iizuka-Hishikawa Y (2017). Lysophosphatidic acid acyltransferase 3 tunes the membrane status of germ cells by incorporating docosahexaenoic acid during spermatogenesis. J. Biol. Chem..

[19] Sato H (2010). Group III secreted phospholipase A2 regulates epididymal sperm maturation and fertility in mice. J. Clin. Invest..

[20] Shishikura K (2019). Acyl-CoA synthetase 6 regulates long-chain polyunsaturated fatty acid composition of membrane phospholipids in spermatids and supports normal spermatogenic processes in mice. FASEB J..

[21] Ren M (2019). Extramitochondrial cardiolipin suggests a novel function of mitochondria in spermatogenesis. J. Cell Biol..

[22] Gulaya NM (2001). Phospholipid composition of human sperm and seminal plasma in relation to sperm fertility. Arch. Androl..

[23] Kotani H, Taimatsu K, Ohga R, Ota S, Kawahara A (2015). Efficient Multiple Genome Modifications Induced by the crRNAs, tracrRNA and Cas9 Protein Complex in Zebrafish. PLoS ONE.

[24] Naito Y, Hino K, Bono H, Ui-Tei K (2015). CRISPRdirect: software for designing CRISPR/Cas guide RNA with reduced off-target sites. Bioinformatics.

[25] Kawana H (1864). An accurate and versatile method for determining the acyl group-introducing position of lysophospholipid acyltransferases. Biochim. Biophys. Acta Mol. Cell Biol. Lipids..

[26] Ohno Y, Kamiyama N, Nakamichi S, Kihara A (2017). PNPLA1 is a transacylase essential for the generation of the skin barrier lipid omega-O-acylceramide. Nat. Commun..

