# Supplementary material for: Abnormal male reproduction and embryonic development induced by downregulation of a phospholipid fatty acid-introducing enzyme Lpgat1 in zebrafish
Source: Sci Rep. 2022 May 4;12:7312. doi: 10.1038/s41598-022-11002-4 (PMC9068807; doi:10.1038/s41598-022-11002-4)
Supplement: Supplementary file 2 — Supplementary Information 1. [file 41598_2022_11002_MOESM2_ESM.pdf]

Scientific Reports

Supplemental Information

Abnormal male reproduction and embryonic development induced by downregulation of a phospholipid fatty acid-introducing enzyme Lpgat1 in zebrafish

Takeaki Shibata, Hiroki Kawana, Yuri Nishino, Yoshiko Ito, Hiroyasu Sato, Hirofumi Onishi, Kuniyuki Kano, Asuka Inoue, Yoshitaka Taketomi, Makoto Murakami, Satoshi Kofuji, Hiroshi Nishina, Atsuo Miyazawa, Nozomu Kono and Junken Aoki

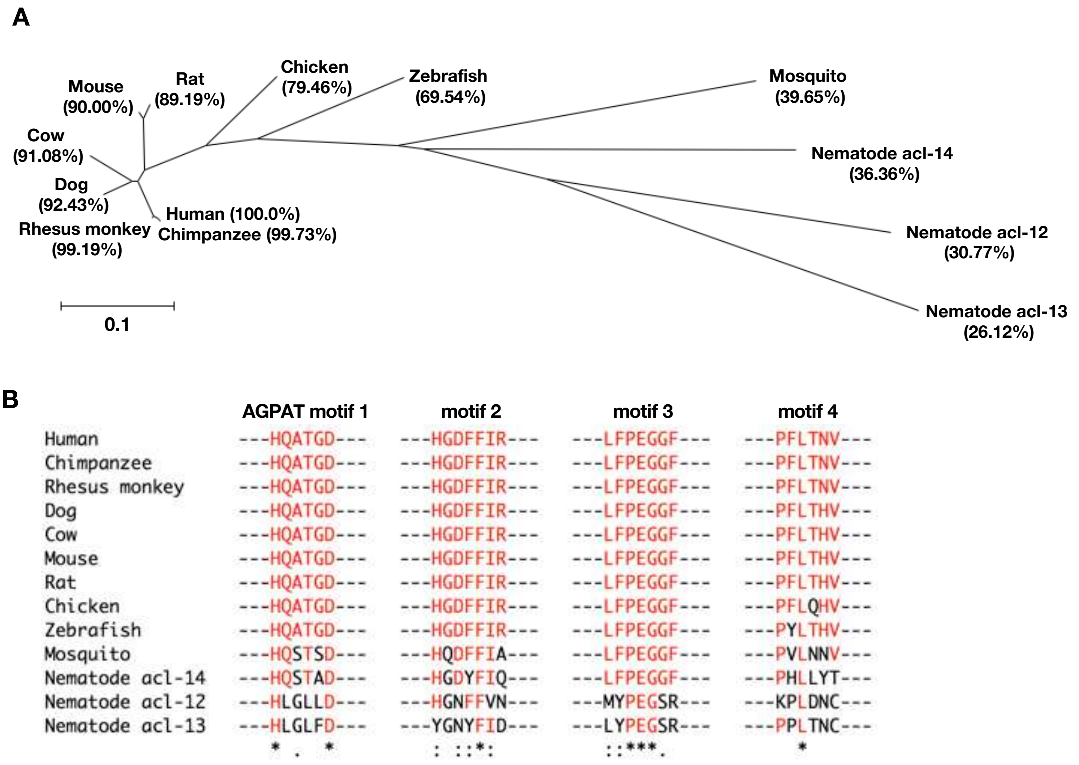

Supplementary Figure S1. Conservation of *LPGAT1* gene among species.

Phylogenetic analysis (**A**) and amino acid sequence alignment of important motifs for enzymatic activity (**B**) of human (*H. sapiens*), chimpanzee (*P. troglodytes*), rhesus monkey (*M. mulatta*), dog (*C. lupus*), cow (*B. taurus*), mouse (*M. musculus*), rat (*R. norvegicus*), chicken (*G. gallus*), zebrafish (*D. rerio*), mosquito (*A. gambiae*), and nematode (*C. elegans*) LPGAT1. (**A**) Amino acid sequence homology with human LPGAT1 is shown in percentage. Scale bar, 10% amino acid difference. (**B**) Conserved amino acids are shown in red letters. "*" indicates an exact amino acid match, ":" indicates strong amino acid similarity, and "." indicates weak amino acid similarity.

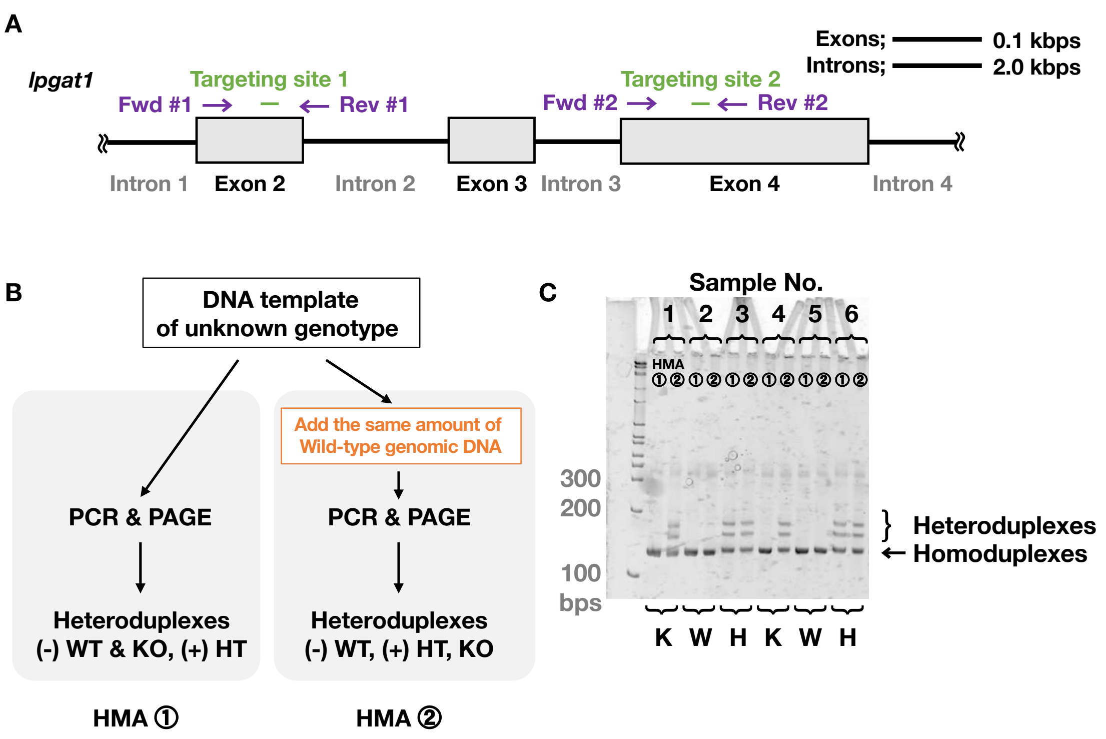

Supplementary Figure S2. Genotyping of *lpgat1* mutants.

(**A**) Schematic drawing of exon-intron structure of zebrafish *lpgat1*. *lpgat1* consists of seven exons, and the gene structure from second to fourth exons is indicated. Exons are shown as gray boxes, and introns as black lines. Two targeting sites shown in Fig. 1A are indicated by the green lines and the sites of primers used for PCR was indicated by purple arrows. (**B**) Strategy for genotyping using PCR and Heteroduplex mobility assay (HMA), which detects small sequence differences, was performed both in the absence (①) or the presence (②) of wild-type genomic DNA, and the amplicons were analyzed by polyacrylamide gel electrophoresis (PAGE). In condition ①, wild-types and homozygous mutants give rise to a single band (homoduplex) and heterozygotes give rise to multiple bands (homoduplex and heteroduplexes). (**C**) Image of a representative gel for determining the genotype of mutant 1. The determined genotypes (as judged by the presence of heteroduplexes) of each sample were shown on the bottom of the figure (W; wild-type, K; homozygous mutants and H; heterozygote).

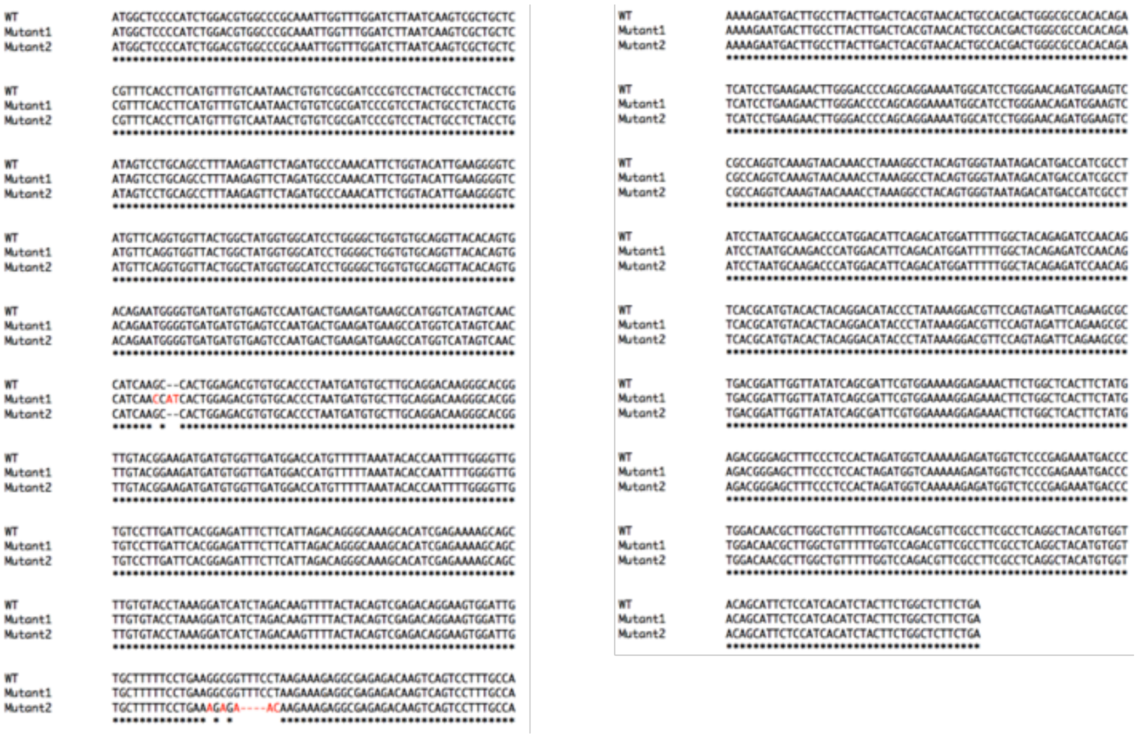

Supplementary Figure S3. The nucleotide sequences of the reverse transcripts (cDNAs) of the mutant 1 and mutant 2 *lpgat1* mRNAs.

The nucleotide sequence of cDNAs encoding mutant 1 and 2 Lpgat1 proteins (ORF) are aligned with that of wild-type (WT) cDNA. "*" indicates a nucleotide match. Mutations are shown in red letters.

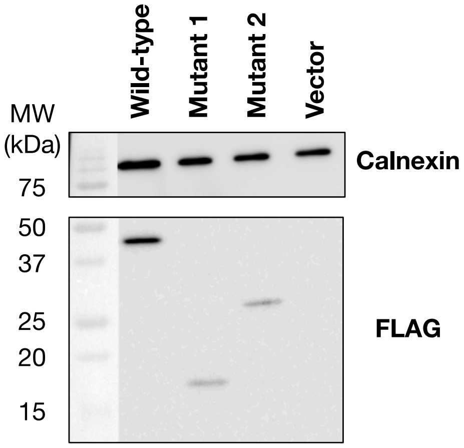

Supplementary Figure S4. Expression of mutant Lpgat1 proteins in HEK293A cells. HEK293A cells were transfected with cDNAs encoding wild-type, mutant 1 or 2 Lpgat1 proteins with FLAG-epitope tag. Immunoblotting was performed using membrane fractions of each cells, anti-FLAG and anti-calnexin (loading control) antibodies.

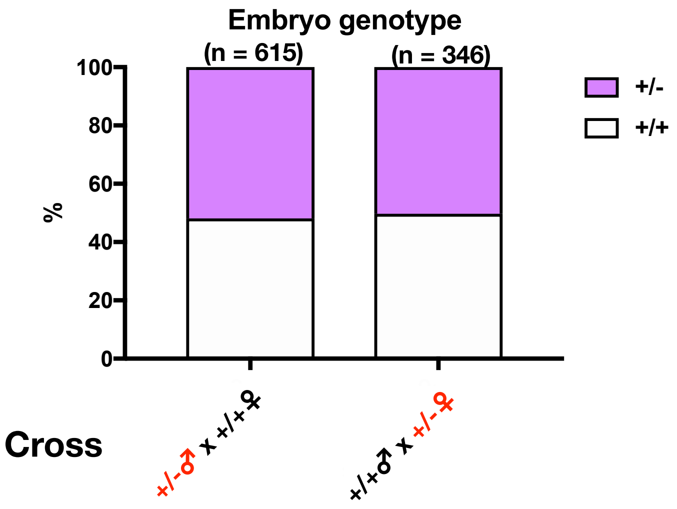

Supplementary Figure S5. Genotype of embryos obtained by crossing heterozygote males and wild-type female.

The genotype of embryos which were obtained by crossing heterozygous males with wild-type females and showed the sign of cleavage was determined. They produced equal proportions of wild-type (+/+) and heterozygote (+/-) (left bar). The result from crossing wild-type males with heterozygous females is also shown (right bar). Embryos were acquired from six independent crosses and showed an integrated value. The numbers of embryos analyzed were indicated on the top of each bar.

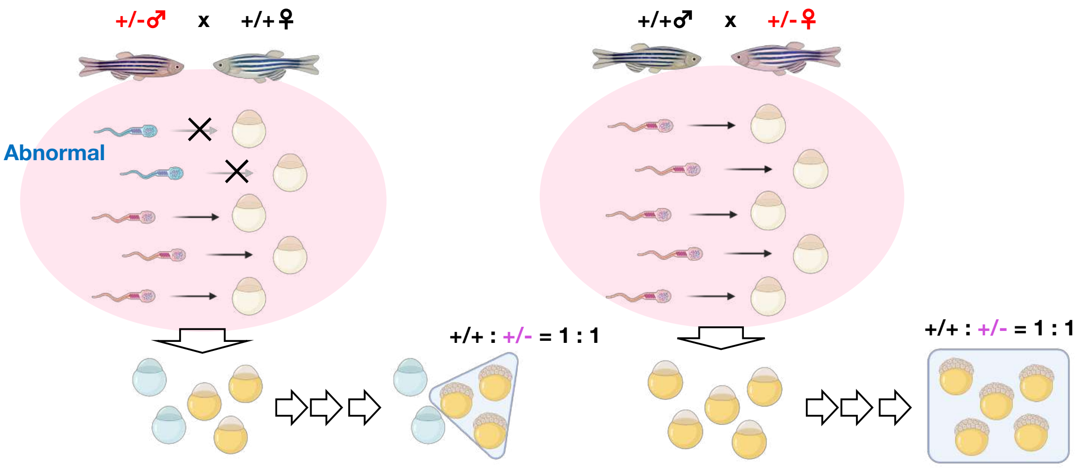

Supplementary Figure S6. Schematic diagram of *lpgat1*$^{+/-}$ male phenotype.
lpgat1$^{+/-}$ males have some, but not all, abnormal sperm, which probably are unable to fuse with ovule, resulting in the appearance of ovule like eggs (shown in blue). Like in wild-type zebrafish, *lpgat1* (-) and *lpgat1* (+) sperm are almost equal in number, resulting the appearance of wild-type (*lpgat1*$^{+/+}$) and heterozygote (*lpgat1*$^{+/-}$) in same proportion. This figure was created with BioRender.com (https://biorender.com)

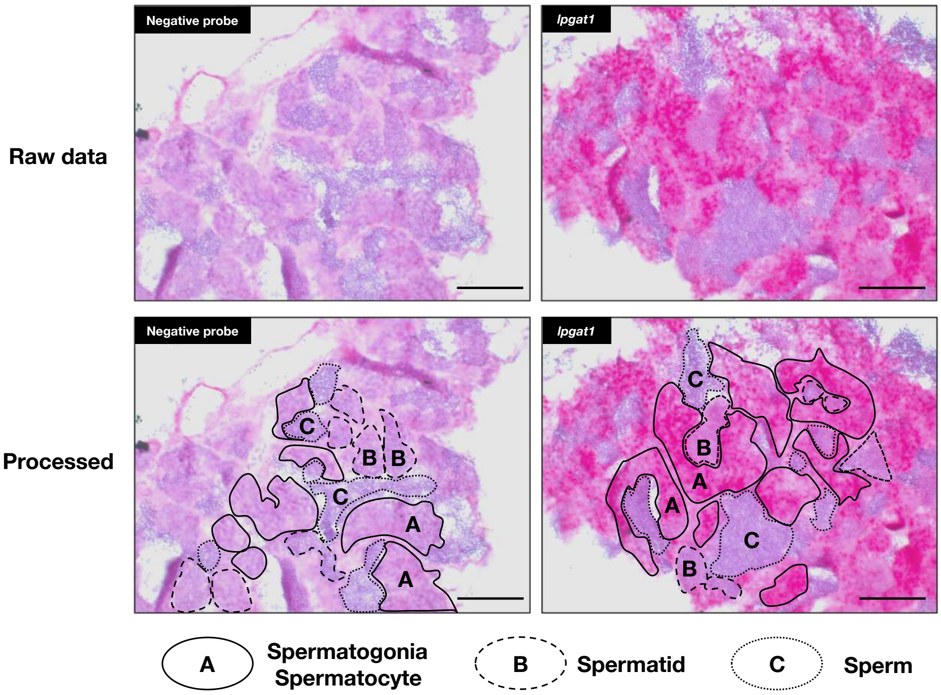

Supplementary Figure S7. RNA *in situ* hybridization of *lpgat1*.

In situ hybridization analysis of *lpgat1* mRNA in adult zebrafish testis. The magenta and purple signals indicate the distribution of *lpgat1* mRNA and nuclei, respectively. The testes were divided into three regions; the areas of spermatogonia and spermatocyte, which are distinguished by their huge nucleus stained with hematoxylin and cytoplasm stained with eosin (A), those of spermatid, which is distinguished by its region with small nucleus stained with hematoxylin and cytoplasm stained with eosin (B), and those of sperm, which are distinguished by their small nuclei stained with hematoxylin and not stained by eosin (C). Scale bar, 100 µm.

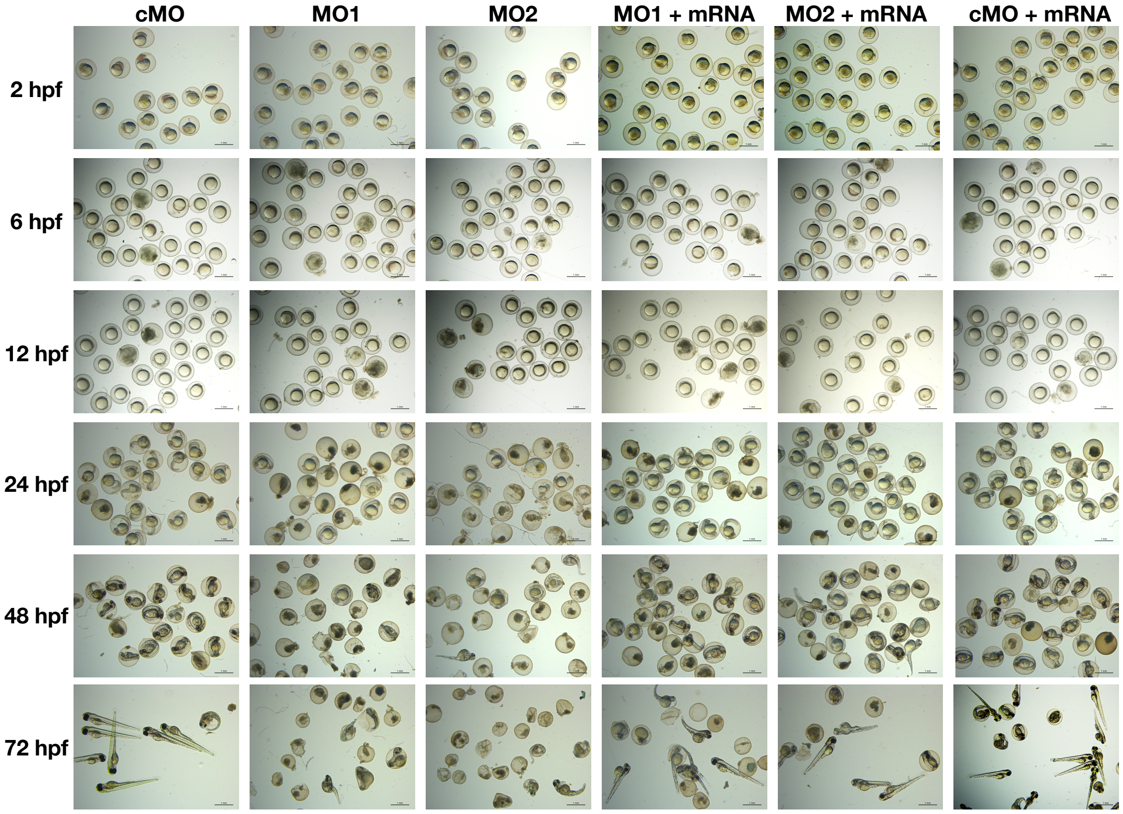

Supplementary Figure S8. Downregulation of *lpgat1* by morpholino oligonucleotides exhibits embryonic developmental defects.

Embryos were injected with morpholino antisense oligonucleotides (MO) specific to *lpgat1* (MO1 and MO2), which bind to the exon-intron junction of immature *lpgat1* mRNA and inhibit the production of mature mRNA, or control MO (cMO) in combination with *lpgat1* mRNA. Representative images of each time point are shown. Scale bar, 1 mm.

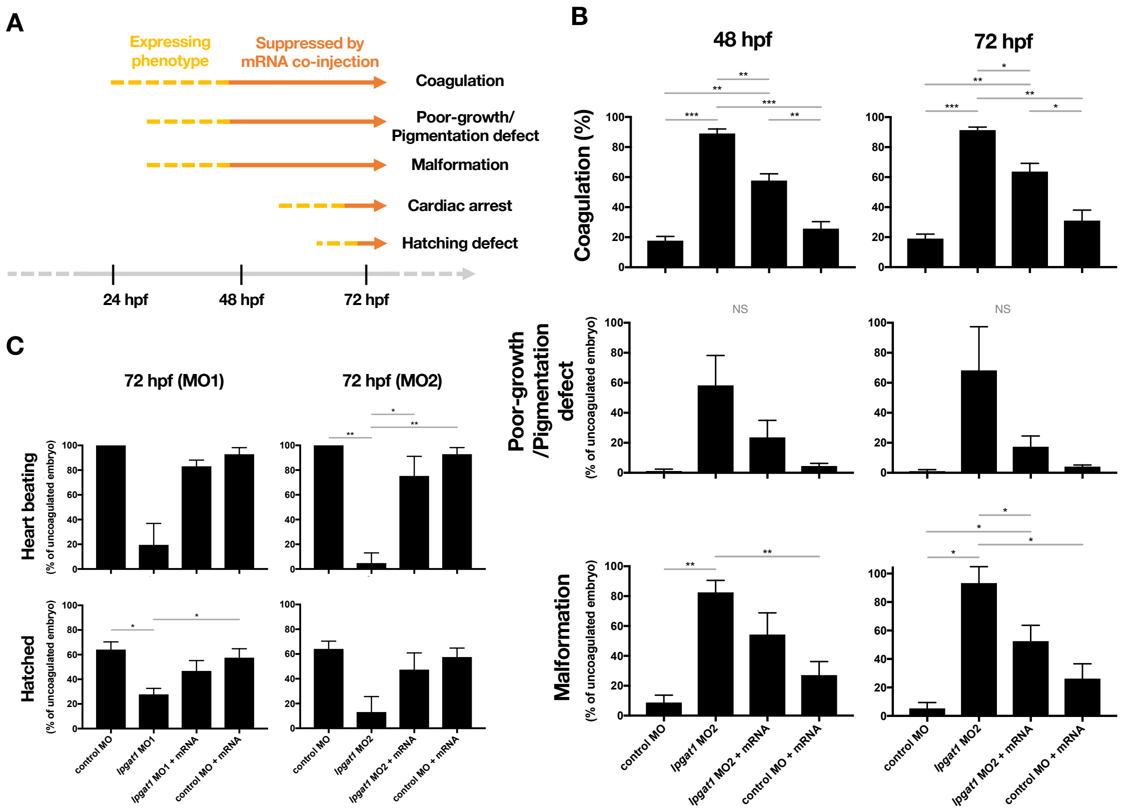

Supplementary Figure S9. MO-induced developmental defects were partially rescued by co-injection of *lpgat1* mRNA.

(**A**) Summary of phenotypes observed by downregulating *lpgat1* in zebrafish over time. (**B**) Upper graphs, percentage of coagulated eggs after injection of morpholino antisense oligo (MO) specific to *lpgat1* (MO2) or control MO (cMO) in combination with *lpgat1* mRNA. MO2 is a splicing inhibitor, which bind to the exon-intron junction of immature *lpgat1* mRNA and inhibit the splicing of pre-mRNA. Middle graphs, percentage of pigmentation defect and bottom graphs, malformation in uncoagulated embryos. hpf; hours post fertilization. (**C**) Injection of MO1 (left) and MO2 (right) induced cardiac arrest (upper graphs) and hatching delay (lower graphs) in survived larva at 72 hpf. Percentages of heart beating larva and hatched larva are shown. The means of the data from three independent experiments for each group with n = 200 in total are shown. Error bars are S.D. Statistically significant differences are marked with asterisk. *$p < 0.05$; **$p < 0.01$, ***$p < 0.001$. Two-way ANOVA, Holm's multiple comparison test was used.

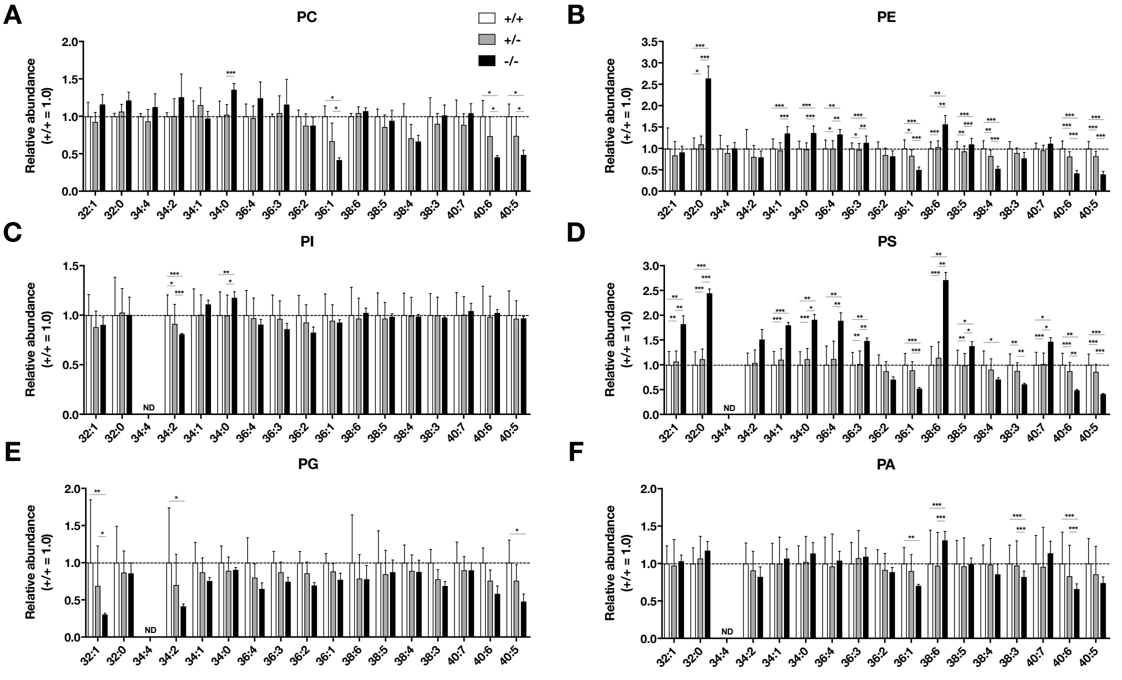

Supplementary Figure S10. Lipidomic analyses of *lpgat1* mutant zebrafish.

Data of Figure 6 was re-analyzed and presented as relative abundance for each phospholipid species. Lipids were collected at 7 days post fertilization from a single zebrafish larva obtained from the crossing between *lpgat1*$^{+/-}$ male and female and analyzed the molecular species for phosphatidylcholine (PC, **A**), phosphatidylethanolamine (PE, **B**), phosphatidylinositol (PI, **C**), phosphatidylserine (PS, **D**), phosphatidylglycerol (PG, **E**), and phosphatidic acid (PA, **F**) by LC-MS/MS. Genotypes of each larva were determined by PCR after lipid sample was collected. The fish are obtained from three independent crosses. Number of samples were n = 18 for *lpgat1*$^{+/+}$ (white bars), n = 19 for *lpgat1*$^{+/-}$ (gray bars) and n = 7 for *lpgat1*$^{-/-}$ (black bars). Error bars are S.D. Statistically significant differences are marked with asterisk. *$p < 0.05$; **$p < 0.01$; ***$p < 0.001$. Two-way ANOVA, Bonferroni's multiple comparison test was used.

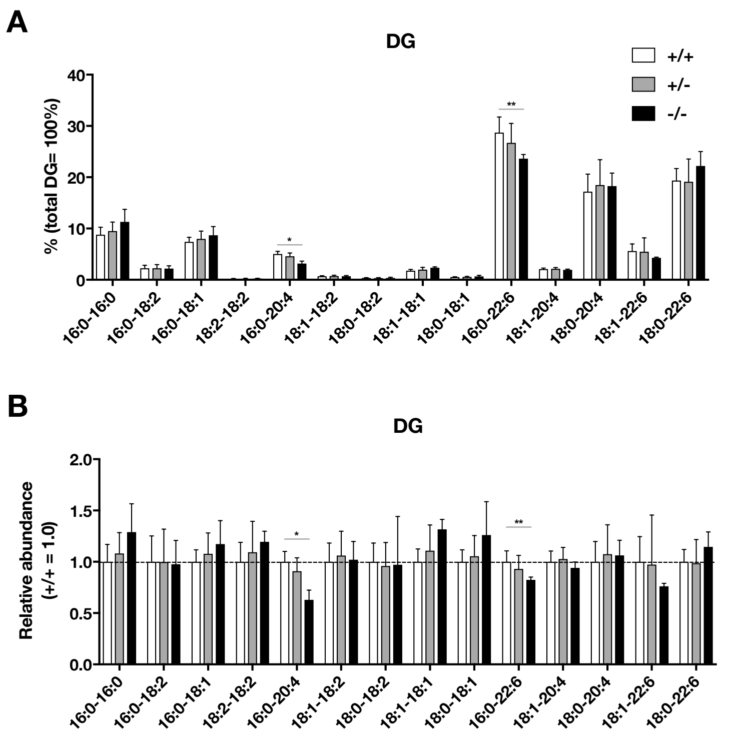

Supplementary Figure S11. Diacylglycerol species in *lpgat1* mutant zebrafish.
Lipids were collected at 7 days post fertilization from zebrafish larvae obtained from the crossing between *lpgat1*$^{+/-}$ male and female and analyzed the molecular species for diacylglycerol (DG, **A**), and the relative abundance of each molecular species are shown (**B**). Number of samples were n = 18 for *lpgat1*$^{+/+}$ (white bars), n = 19 for *lpgat1*$^{+/-}$ (gray bars) and n = 7 for *lpgat1*$^{-/-}$ (black bars). Error bars are S.D. Statistically significant differences are marked with asterisk. *p < 0.05; **p < 0.01. Two-way ANOVA, Bonferroni's multiple comparison test was used.

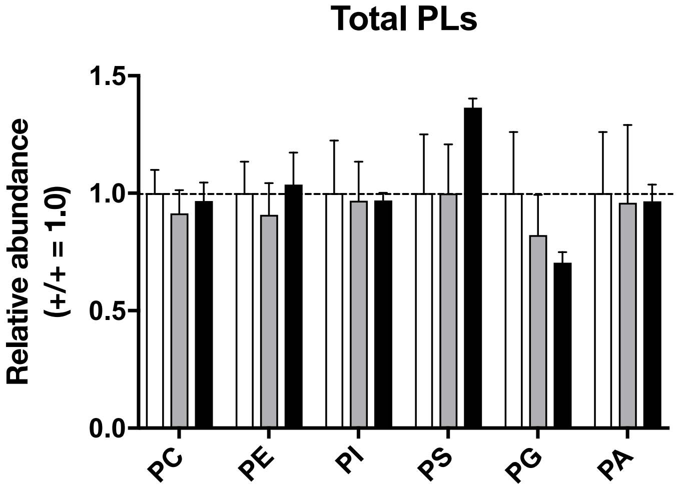

Supplementary Figure S12. Total phospholipids in *lpgat1* mutant zebrafish.

Total amount of phospholipids (PLs) was compared between genotypes based on the sum of the area ratio values of each phospholipid molecular species. Number of samples were n = 18 for *lpgat1*$^{+/+}$ (white bars), n = 19 for *lpgat1*$^{+/-}$ (gray bars) and n = 7 for *lpgat1*$^{-/-}$ (black bars). Error bars are S.D. Statistically significant differences are marked with asterisk. *$p < 0.05$; **$p < 0.01$. Two-way ANOVA, Bonferroni's multiple comparison test was used.

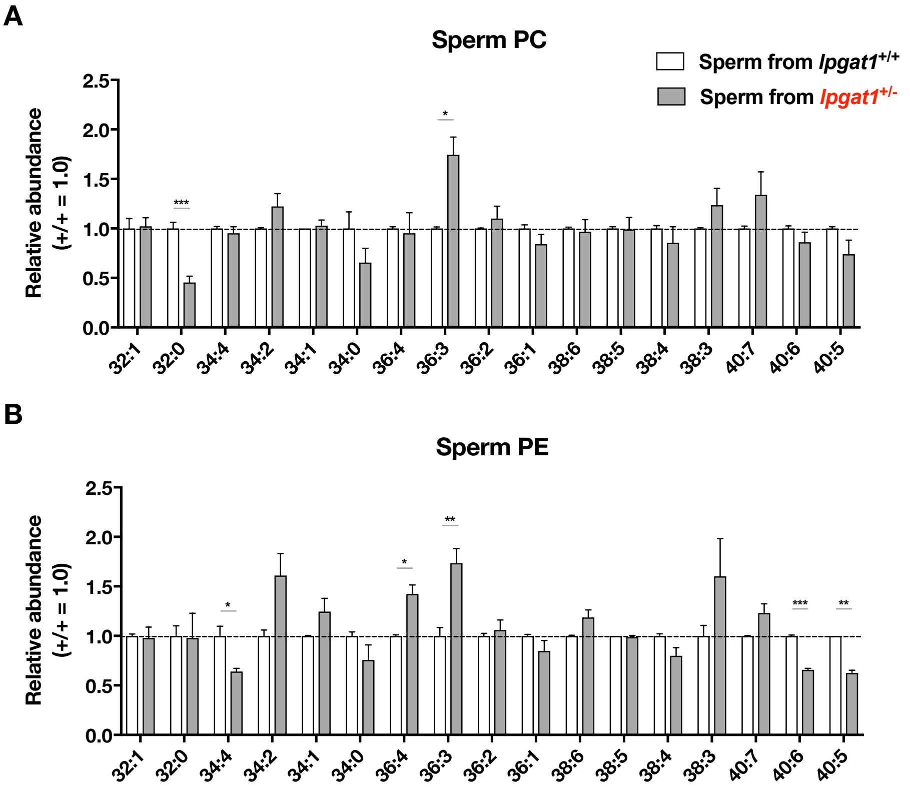

Supplementary Figure S13. Phosphatidylcholine (PC) and phosphatidylethanolamine (PE) species in sperm from *lpgat1*$^{+/-}$ zebrafish.

Data of Figure 7 was re-analyzed and presented as relative abundance for each phospholipid species. PC (**A**) and PE (**B**) species composition of zebrafish sperm was analyzed by LC-MS/MS. The relative abundance of each molecular species is shown. Semen was collected from anesthetized zebrafish by abdominal compression. Sperm pellets were prepared from semen collected from one zebrafish and lipids were collected. Number of sperm samples were n = 4 for *lpgat1*$^{+/+}$ (white bars) and n= 4 for *lpgat1*$^{+/-}$ (gray bars). Error bars are S.D. Statistically significant differences are marked with asterisk. *$p < 0.05$; **$p < 0.01$; ***$p < 0.001$. Unpaired, two-tailed t test was used.
